# Interspecific variations in the gastrointestinal microbiota in penguins

**DOI:** 10.1002/mbo3.66

**Published:** 2013-01-25

**Authors:** Meagan L Dewar, John P Y Arnould, Peter Dann, Phil Trathan, Rene Groscolas, Stuart Smith

**Affiliations:** 1 School of Exercise and Nutritional Sciences, Deakin UniversityBurwood, Australia; 2 School of Life and Environmental Sciences, Deakin UniversityBurwood, Australia; 3 Research Department, Phillip Island Nature ParksPhillip Island, Victoria, 3922, Australia; 4 British Antarctic Survey, High CrossMadingley Road, Cambridge, CB3 0ET, U.K; 5 Centre d'Ecologie et Physiologie Energétiques, Centre National de la Recherche Scientifique (CNRS)67087, Strasbourg, France; 6 Molecular and Medical Strategic Research Centre, Deakin UniversityBurwood, Australia

**Keywords:** Microbiota, penguins, pyrosequencing, qPCR

## Abstract

Despite the enormous amount of data available on the importance of the gastrointestinal (GI) microbiota in vertebrate (especially mammals), information on the GI microbiota of seabirds remains incomplete. As with many seabirds, penguins have a unique digestive physiology that enables them to store large reserves of adipose tissue, protein, and lipids. This study used quantitative real-time polymerase chain reaction (qPCR) and 16S rRNA gene pyrosequencing to characterize the interspecific variations of the GI microbiota of four penguin species: the king, gentoo, macaroni, and little penguin. The qPCR results indicated that there were significant differences in the abundance of the major phyla Firmicutes, Bacteroides, Actinobacteria, and Proteobacteria. A total of 132,340, 18,336, 6324, and 4826 near full-length 16S rRNA gene sequences were amplified from fecal samples collected from king, gentoo, macaroni, and little penguins, respectively. A total of 13 phyla were identified with Firmicutes, Bacteroidetes, Proteobacteria, and Fusobacteria dominating the composition; however, there were major differences in the relative abundance of the phyla. In addition, this study documented the presence of known human pathogens, such as *Campylobacter, Helicobacter, Prevotella, Veillonella, Erysipelotrichaceae, Neisseria,* and *Mycoplasma*. However, their role in disease in penguins remains unknown. To our knowledge, this is the first study to provide an in-depth investigation of the GI microbiota of penguins.

## Introduction

Penguins are a distinctive group of flightless seabirds found exclusively in the southern hemisphere, occupying an extensive geographical range extending from the Galapagos Islands to the Antarctic continent (Stonehouse 1975). Penguins, like all seabirds, spend most of their lives at sea, only coming to land to breed and molt (Stonehouse 1975; Reilly 1994; Roeder et al. 2002). Penguins have a unique digestive physiology that enables them to store large amounts of undigested food, build up large reserves of adipose tissue (fat), and store large amounts of protein and lipids for long periods of fasting during breeding and molting (Stonehouse 1975; Reilly 1994; Roeder et al. 2002). The gastrointestinal (GI) tract contains a diverse and complex microbial ecosystem made up of hundreds of different species of microorganisms, which has coevolved with its host (Collins et al. 1994; Koutsos and Arias 2006; Lumpkins et al. 2008; Torok et al. 2008). The GI microbiota has a profound influence on the nutritional, physiological, immunological, and metabolic processes of the host (Mackie et al. 1999; Zoetendal et al. 2004a,b; Musso et al. 2011) playing a significant role in energy harvest, fat metabolism, secretion and synthesis of nutrients, vitamins, amino acids, and the production of short-chain fatty acids from the diet consumed by the host (Collins et al. 1994; Suau et al. 1999; Flint et al. 2007; Torok et al. 2008). Rawls et al. (2004, 2006) identified that in the absence of microbial colonization, the GI tract results in an immature and arrested differentiation. Furthermore, research has shown that the absence of a GI microbiota reduces an animal's ability to secrete and absorb essential vitamins and nutrients from their diet (Penders et al. 2006; Blaut and Clavel 2007).

To date, the mammals and poultry GI microbiota have been extensively studied and shown to be dominated by two main phyla: Firmicutes (usually 60–80% of total composition) and Bacteroidetes (usually 20–40% of total composition) in multiple vertebrate species including mammals (such as humans, rodents, and pinnipeds), poultry, and livestock (i.e., cattle and pigs). (Ley et al. 2005; Gabriel et al. 2006; Ley et al. 2006a,b, 2008a,b; Glad et al. 2010a,b). The avian microbiota, however, comprise approximately 640 species from 140 genera, with only 10% of the avian microbiota being cultured in the laboratory (Torok et al. 2008). However, much of what we know about the avian microbiota is based from research on poultry (DeGolier et al. 1999; Zhu et al. 2002; Apajalahti and Kettunen 2005).

Despite the enormous amount of data available on the importance of microbes in mammals, it is surprising that so little research has been carried out on avian species, with the exception of poultry (chicken, turkey) (Zhu et al. 2002; Apajalahti and Kettunen 2005). To date, the composition and role of this ecosystem remain incomplete for many seabird species, including penguins, with earlier studies being based on the use of culture dependant techniques to provide description on the composition and abundance of members of the microbial community (Potti et al. 2002; Thouzeau et al. 2003; Zoetendal et al. 2004a,b; Bonnedahl et al. 2005) or to look for specific microbial pathogens, such as *Salmonella* (Olsen et al. 1996; Palmgren et al. 2000), *Campylobacter* (Quessy and Messier 1992; Broman et al. 2000; Hubalek 2004; Bonnedahl et al. 2005; Leotta et al. 2006a,b; Griekspoor et al. 2009), and *Pasteurella multocida* (DeLisle et al. 1990; Leotta et al. 2003; Weimerskirch 2004; Leotta et al. 2006a,b). However, these techniques do not accurately reflect the actual microbial composition but only those that can be cultured using selective media and as a result, providing an inaccurate account on the composition and abundance of microbes from complex biological systems such as the GI tract. Therefore, earlier studies on microbial composition of penguins may be considered incomplete. For this reason, many microbiologists have turned to molecular methods, such as quantitative real-time polymerase chain reaction (qPCR) and 16S rRNA gene pyrosequencing, to characterize and explore the microbial composition of complex ecosystems, such as the GI tract, ocean, and soil. These methods are extensively used in studies of humans and other vertebrate species (predominantly livestock and poultry). For penguins, the use of molecular-based methods to examine the microbial composition is limited to two studies: Zdanowski et al. (2004) and Banks et al. (2009). Zdanowski et al. examined the microbial composition of freshly deposited Adélie penguin guano, whereas Banks et al. examined the influence of geographical separation and host phylogeny on the microbial composition of Adélie penguins. Both studies documented that Adélie penguins are highly dominated by Firmicutes (41%), Actinobacteria (35%), and Proteobacteria (12.5%), while Banks et al. (2009) also noted the presence of Bacteroidetes (5%). In addition, Banks et al. (2009) documented that a negative correlation exists between host relatedness and microbial community similarity and no significant correlation between geographical location and microbial composition, indicating that host phylogeny influences the microbial composition of an individual.

Characterization of the vastly diverse ecosystem of the GI tract is the first step in exploring its role in digestive physiology, health, and disease (Eckburg et al. 2005). Therefore, this study aims to use quantitative real-time PCR and 16S rRNA, sequencing to characterize the microbial composition and diversity of the fecal microbiota of four species of penguins.

## Materials and Methods

### Study species, sites, and sample collection

Fecal samples were collected on land from king (*Aptenodytes patagonicus*) (*n* = 12), gentoo (*Pygoscelis papua*) (*n* = 12), macaroni (*Eudyptes chrysolophus*) (*n* = 12), and little penguin (*Eudyptula minor*) (*n* = 12) returning from foraging trips during the breeding season at three different study sites, including Bird Island, South Georgia (54°00′S, 38°03′W); Baie du Marine, Possession Island Crozet Archipelago (46°25′S, 51°52 E); and Phillip Island, Australia (38.4833°S, 145.2333°E). Birds were collected when returning to their nests, with fecal samples obtained by rectal swab (Copan Eswabs, Copan™, Brescia, Italy) and stored in liquid nitrogen for transport.

### Sample analysis

#### DNA extraction and real-time PCR

DNA was extracted using the Qiagen™ QIAamp DNA Stool Mini Kit (Hilden, Germany) following the manufacturer's instructions. The major phyla selected for qPCR analysis were chosen from previous studies that had examined the predominant GI microbiota of vertebrates (mammals, chickens, and Adélie penguins) (Richberg 2000; Blackwood et al. 2005; Eckburg et al. 2005; Ley et al. 2005, 2008a,b; Banks et al. 2009). The phyla tested in this study were Firmicutes, Bacteroidetes, Actinobacteria, and Proteobacteria. The primer sequences and annealing temperature for the chosen bacterial groups can be found in the Table S1. The quantitative real-time PCR was performed on the Stratagene MX3000P. Each PCR mixture comprises 5 μL of Brilliant II SYBR green (Stratagene™, La Jolla, CA), 20 pmol/μL of forward and reverse primer, 2 ng of template DNA and made up to a final volume of 20 μL with nuclease-free water. The cycling conditions were 95°C for 2 min, followed by 40 cycles of 95°C for 5 sec, followed by annealing temperature (listed in Table S1) for 30 sec with all samples run in triplicate. Bacterial concentration was determined by comparing the threshold value (ct values) with a standard curve. The standard curve was created by using a serial 10-fold dilution from DNA extracted from a pure culture of *Escherichia coli* ranging from 10^4^ to 10^10^ colony-forming units (CFU)/g.

### PCR amplification of 16S ribosomal RNA gene sequences

#### Gentoo, little, and macaroni penguin samples

From the original samples, four individuals per species were chosen for 16S rRNA pyrosequencing. All samples/species were pooled together with the attachment of MID tag barcodes (i.e., Barcode 338R_BC0495 “TCACTGGCAGTA” was attached to all little penguin samples). Samples were then amplified using universal primers Roche adapter A (5′-GCC TCC CTC GCG CCA TCA GT-3′) and reverse 338R (5′-CAT GCT GCC TCC CGT AGG AGT-3′) to amplify the V2–V3 region. Following amplification, samples were sequenced on the Roche/454 GS FLX Titanium Genome Sequencer (Roche Diagnostic Corporation, Basel, Switzerland) by Engencore (USA) according to Fierer et al. (2008). All sample preparation and sequencing were performed by Engencore according to the Roche 454 and Fierer et al. (2008) protocol. Following sequencing, barcodes were removed using Roche SFF software (Roche Applied Science, Indianapolis, IN).

#### King penguin samples

Results from an unpublished study on the microbial composition of king penguins were included in this study for comparison. In the previous study, the V2–V3 region was amplified using primers 8F (5′-AGAGTTTGATCCTGG-3′) (Lane 1991) and 519R (5′-TTACCGCGGCTGCT-3′) (Felske et al. 1997). Following PCR amplification, samples were pooled and run on the Roche 454 GS FLX Titanium genome sequencer at CSIRO.

### Sequence analysis

Quality control, removal of chimera's, sequence alignment, identification, and operational taxonomic unit (OTU) classification were performed by Ribocon GmbH (Germany) as per the protocol of Prausse et al. (2007, 2012). The 16S rRNA sequences reported in this study have been submitted to EMBL under accession number ERP001504.

### Statistical analysis

To determine if there were significant differences between all penguin species for the major phyla for qPCR analysis, a one-way analysis of variance (ANOVA), with Tukey's HSD (Honesty Significant Difference) for pairwise comparison, was performed in SPSS (IBM Corportation, Armonk, NY) and was considered significantly different if *P* < 0.05. A multidimensional scaling plot (MDS), species diversity (Shannon–Weiner), and cluster analysis were performed on both the qPCR and pyrosequencing data using Primer E version 6, Methodology as per Clarke (1993). The samples that share the highest similarity will be represented on the plot by points plotted closest together, whereas samples that share limited similarity will be represented on the plot by points that are plotted farthest apart (Richberg 2000).

Chi-squared analysis performed in Calypso version 3 was used to analyze the statistical difference between gentoo, little, and macaroni penguins, although statistical power was quite low due to pooling of samples (Table S2). The samples from king penguins could not be included in the statistical analysis due to the differences in sequencing protocol and primers used.

### Diversity indices

Microbial diversity for the 16S rRNA data for all penguin species was calculated by Shannon–Weiner diversity index (H') using Primer E. This index was calculated by the following equation:


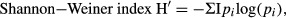


where *p*_*i*_ is the proportion of the total count arising from the *i*th species.

## Results

### Quantitative real-time PCR

**Figure 1:**
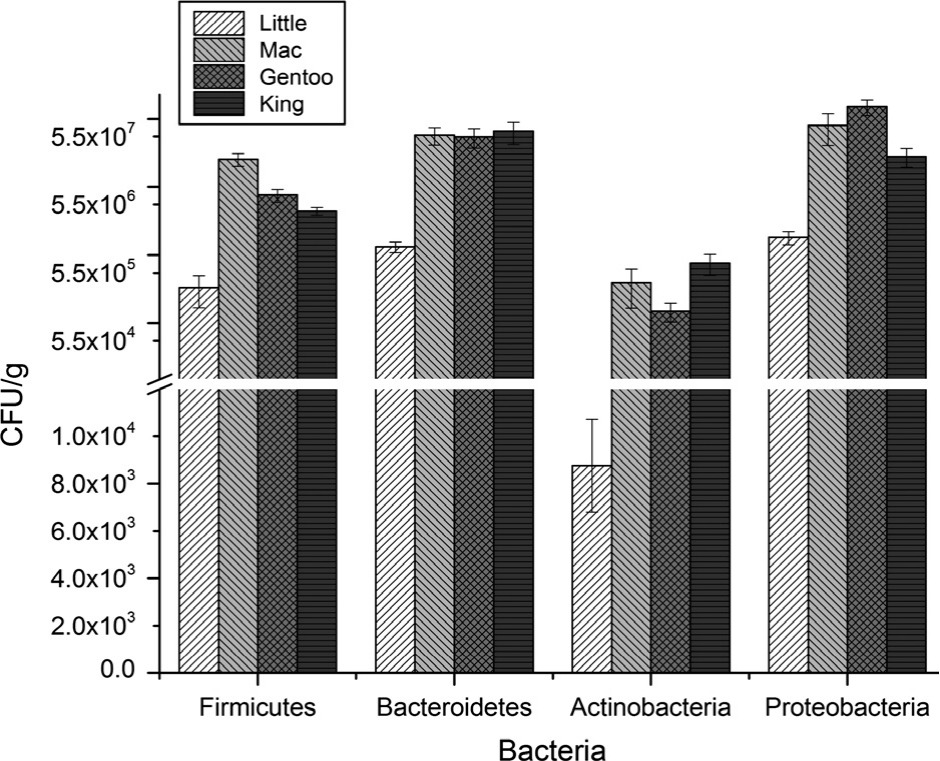
The abundance of all major phyla are significantly different for all penguin species (*P* < 0.000). The abundance of each phylum was measured using quantitative real-time polymerase chain reaction (PCR) and phylum-specific primers.

The abundances of Firmicutes, Bacteroidetes, Proteobacteria, and Actinobacteria were detected from DNA extracted from fecal samples of king, gentoo, macaroni, and little penguins and using phyla-specific primers. The CFU value for little penguins was significantly lower than all other penguin species for all major phyla (ANOVA, *P* = 0.001). Macaroni penguins had a significantly higher abundance of Firmicutes in comparison with king (ANOVA, *P* = 0.002) and gentoo (ANOVA, *P* = 0.008) penguins. Significant differences in the abundance of king and gentoo penguins were observed for the phyla Proteobacteria (ANOVA, *P* = 0.041). However, there were no significant differences between macaroni and king and macaroni and gentoo penguins for Proteobacteria. For Actinobacteria, there were no significant differences observed between macaroni, king, and gentoo penguins (Fig. 1).

**Figure 2:**
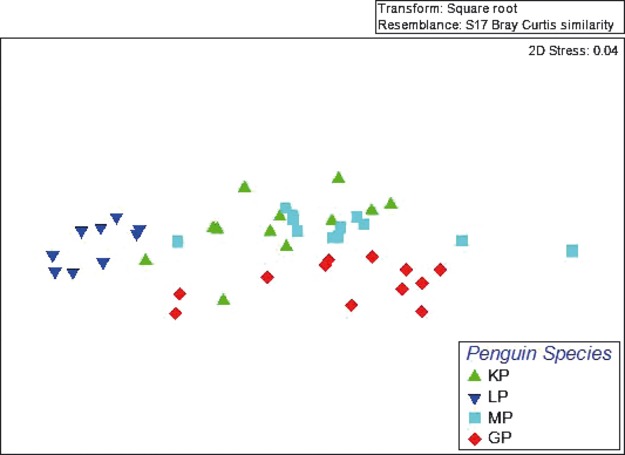
Similarity of the major bacterial phyla of penguin species using quantitative polymerase chain reaction (qPCR).

The results from the MDS analysis show evidence that individual little penguins are clustering together, indicating limited variation within this species. Some individual gentoo, king, and macaroni penguins appear to be clustered together, while other individuals appear to be separated, indicating similarities between the three penguin species and also the potential for individual variation, within each species (Fig. 2).

### 16S rRNA gene pyrosequencing

#### Microbial composition of penguins

Results from the 16S rRNA pyrosequencing for each population were pooled together to characterize the microbial composition of each population. A total of 132,340, 18,336, 6324, and 4826 near full-length 16S rRNA gene sequences were amplified from fecal samples collected from king, gentoo, macaroni, and little penguins, respectively. With 97% sequence similarity, a total of 1331, 2195, 1362, and 561 OTUs were identified, respectively.

**Figure 3:**
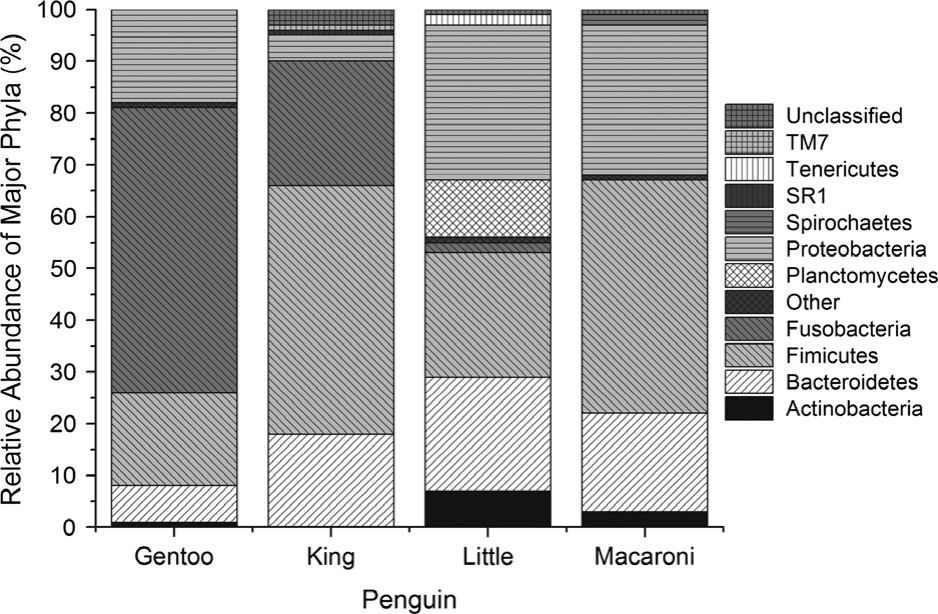
Firmicutes, Bacteroidetes, and Proteobacteria dominate the microbial composition of all penguin species. While Fusobacteria, is also dominant in gentoo and king penguins. However, the composition of the microbiota differs between penguins. Fecal DNA was amplified using genetic primers targeting the V2–V3 region by polymerase chain reaction (PCR). PCR amplicons were then sequenced on the GS FLX Titanium Sequencer.

From the phylogenetic analyses, there were 13 classified phyla represented in penguin gut microbiota: Firmicutes, Bacteroidetes, Proteobacteria, Fusobacteria, Actinobacteria, Chloroflexi, Cyanobacteria, Deferribacteres, Deinococcus-Thermus, Fibrobacteres, Planctomycetes, Spirochetes, and Synergistetes, and four recently classified candidates (BD1-5; OP10, SR1, and TM7) were also represented. The most abundant phyla present in all penguin populations included the Bacteroidetes, Firmicutes, Proteobacteria, and Fusobacteria. Although all four species of penguin show a partial overlap of highly abundant phyla, there are differences in the abundance of each phylum between all four penguin species. Gentoo penguins were dominated by Fusobacteria (55%), Firmicutes (18%), Proteobacteria (18%), and Bacteroidetes (7%). In king penguins, Firmicutes (48%), Fusobacteria (24%), and Bacteroidetes (18%) dominate. The dominant phyla in little penguins were Proteobacteria (30%), Firmicutes (24%), Bacteroidetes (22%), Planctomycetes (11%), and Actinobacteria (7%). While in macaroni penguins, Firmicutes (45%), Proteobacteria (29%), and Bacteroidetes (19%) dominate (Fig. 3).

At the family level, gentoo penguins were highly dominated by Fusobacteriaceae (55%), Moraxellaceae (6%), Leuconostocaceae (6%), Lachnospiraceae (4%), Streptococcaceae (4%), and Flavobacteriaceae (3%). In king penguins, Leuconostocaceae (19%), Campylobacteriaceae (11%), Porphyromonadaceae (11%), Helicobacteriaceae (8%), Flavobacteriaceae (8%), Moraxellaceae (7%), and Streptococcaceae (7%) are the dominant families. Macaroni penguins are heavily dominated by Leuconostocaceae (31%) followed by Porphyromonadaceae (15%), Moraxellaceae (13%), Neisseriaceae (6%), Streptococcaceae (3%), and Lachnospiraceae (3%). While in little penguins, the dominant families were Enterobacteriaceae (16%), Porphyromonadaceae (13%), Phycisphaeraceae (11%), Lactobacillaceae (7%), Bacteroidaceae (4%), Streptococcaceae (3%), Moraxellaceae (3%), Neisseriaceae (3%), Comamonadaceae (3%), Lachnospiraceae (3%), and Leuconostocaceae (3%).

**Figure 4:**
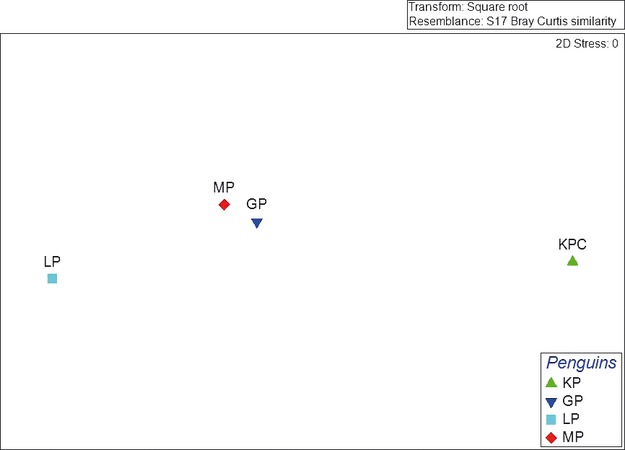
Similarity of the gut microbiota of penguin species from 16S rRNA pyrosequencing data. There is a high level of similarity between gentoo and macaroni penguins, as indicated by the close clustering. For king and little penguins, there is a low level of similarity to other penguin species.

MDS analysis is used to examine the similarity between samples, with highly similar samples clustering closely together on the plot, whereas samples of limited similarity are further apart on the plot. The MDS analysis of the penguin microbiota demonstrates a high level of similarity between gentoo and macaroni penguins. For king and little penguins, there is little similarity with other penguin species (Fig. 4). The analysis of similarity results also indicates a high level of similarity between gentoo and macaroni penguins, sharing approximately 50% of the microbial species. Little penguins share approximately 40% of their microbiota with gentoo and macaroni penguins, whereas king penguins have the lowest level of similarity, sharing less than 10% of their microbiota with the other penguin species (Fig. S1).

#### Actinobacteria

Actinobacteria had the lowest abundance of the major microbial phyla with less than 1–7% of sequences. Only two families are present in all four penguin species Actinomycetaceae and Corynebacteriaceae, with abundances varying between 10–46% and 14–41%, respectively. Propionibacteriaceae is prevalent in gentoo (20%), little (18%), and macaroni penguins (12%), but absent from king penguins. In addition, Dermatophilaceae is found to be a major component of Actinobacteria in both king (20%) and gentoo (9%) penguins, but absent in little and macaroni. Although there are similarities in composition, the proportions of these families vary between penguin species (Fig. S1).

#### Bacteroidetes

All four penguin species are highly dominated by Porphyromonadaceae ranging from 45% to 91% of the total composition of the phylum Bacteroidetes. The remainder of the composition greatly varies from species to species with Flavobacteriaceae (48%) dominating gentoo penguins, and Bacteroidaceae (19%), Prevotellaceae (8%), and Unclassified bacteria (9%) in little penguins. In king and macaroni, the remaining 9% and 14% were made up of Flavobacteriaceae and unclassified bacteria in king penguins and Flavobacteriaceae, Rikenellaceae, Chitinophagaceae, Cytophagaceae, and Prevotellaceae in macaroni penguins (Fig. S2).

#### Firmicutes

The largest variation in microbial composition for all penguins occurs within the phyla Firmicutes. In gentoo penguins, Leuconostocaceae (35%), Lachnospiraceae (23%), and Streptococcaceae (20%) dominate. In king penguins, Clostridiales Family XI (40%), Mycoplasmataceae (25%), Peptostreptococcaceae (10%), and Clostridiaceae (9%) are the dominant families. In little penguins, Lactobacillaceae (27%), Streptococcaceae (14%), Leuconostocaceae (11%), Erysipelotrichaceae (11%), and Peptostreptococcaceae (9%) dominate the phyla Firmicutes, whereas for macaroni penguins, 66% of the phyla Firmicutes comprises members from the family Leuconostocaceae, followed by Streptococcaceae (11%) and Lachnospiraceae (7%) (Fig. S3).

#### Proteobacteria

Members of the phylum Proteobacteria were highly dominant in gentoo (18%), little (30%), and macaroni penguins (29%), whereas its dominance was relatively low in king penguins (5%). The major families within this phyla varied among the four penguin species with Moraxellaceae dominating gentoo (43%) and macaroni (44) penguins, Campylobacteriaceae (43%) dominating king penguins, and Enterobacteriaceae (54%) dominating little penguins. Other major families included Pseudomonadaceae (2–7%), Helicobacteriaceae (1–11%), and Neisseriaceae (3–21%) (Fig. S4).

#### Diversity

Diversity indices were calculated for total microbial composition and for each major phylum for each penguin species using the Shannon–Weiner index in Primer E. For total microbial composition, macaroni penguins had the highest microbial diversity with H′ = 3.3, respectively, followed by king, little, and gentoo penguins with a high diversity index of 2.9, 2.5, and 2.4, respectively.

#### Diversity at phylum level

Within the phylum Actinobacteria, the diversity index ranged from 0.88 to 2.3, with king penguins displaying the lowest level of diversity and gentoo penguins displaying the highest. Within the phylum Bacteroidetes, the diversity index ranged from 1.1 to 2.3 with again king penguins displaying the lowest level of diversity and gentoo's displaying the highest. Little penguins had the highest level of diversity for the phylum Firmicutes with a diversity index of 2.67, whereas the macaroni penguins displayed the lowest level of diversity (2.634). Within the phylum Proteobacteria, gentoo and little penguins displayed a high level of diversity with values of 2.77 and 2.32, respectively. King and macaroni penguins displayed an extremely low level of diversity (0.97 and 0.6). All penguin species experienced extremely low levels of diversity for the phyla Fusobacteria ranging from 0.043 to 0.

## Discussion

Prior to this study, limited information was available on the microbial composition and diversity of the penguin microbiota and the variation that exists between different species. In this study, the microbial composition of king, gentoo, macaroni, and little penguins was characterized using qPCR and 16S rRNA amplicon pyrosequencing. qPCR was used to quantify the abundance of specific phylogenetic groups and to examine the influence of individual variation, while the 16S rRNA amplicon sequencing was used to provide a more in-depth characterization of the microbial diversity of four different penguin species.

### Quantitative real-time PCR

The results from the qPCR demonstrated that there was significant variation among all penguin species. In gentoo, macaroni, and little penguins, Proteobacteria appears to be the most dominant phyla with an abundance of 1.5 × 10^8^, 8.0 × 10^7^, and 1.82 × 10^6^ CFU/g, respectively. While in king, Bacteroidetes is the most abundant phyla present with an abundance of 6.6 × 10^7^ CFU/g. Actinobacteria had the lowest abundance level for all penguin species, ranging from 8.77 × 10^3^ to 7.67 × 10^5^ in king penguins.

The MDS results indicate that there is some clustering within the different species, with majority of individuals for each species clustering together, indicating that variation in the microbial composition of different penguin species does exist (Fig. 2). However, the clustering of individuals within each species does not appear to be tight and therefore could indicate individual variation within each species (Fig. 2).

### Influence of “primer sequences” on pyrosequencing results

In this study, we included data from a previously unpublished study on the microbial composition of king penguins (M. L. Dewar et al., unpubl. data) and although the primer sequence used to amplify the V2–V3 region analysis of the sequence data produced has shown that the primer sets used in this study have a similar coverage of the bacterial taxa present in all penguin species as shown in Table S3. Thus, we feel that the sequences used in the king penguin study do not influence the variation in microbial composition between king penguins and the other penguin species.

### 16S rRNA gene pyrosequencing

From the 161,826 sequence reads, a total of 5449 OTUs were identified from all penguin species, covering 13 classified phyla represented in penguin gut microbiota: Firmicutes, Bacteroidetes, Proteobacteria, Fusobacteria, Actinobacteria, Chloroflexi, Cyanobacteria, Deferribacteres, Deinococcus-Thermus, Fibrobacteres, Planctomycetes, Spirochetes, and Synergistetes, and four recently classified candidates (BD1-5; OP10, SR1, and TM7), with Firmicutes, Bacteroidetes, Proteobacteria, and Fusobacteria dominating the penguin microbiota. There were significant differences in the results from the qPCR and 16S rRNA pyrosequencing, with the qPCR results indicating that Bacteroidetes and Proteobacteria were the most dominant phyla in all four species of penguins, while the pyrosequencing has identified that the proportion of Firmicutes and Bacteroidetes is more dominate than Proteobacteria. These differences could be due to the qPCR primer sets being designed based on data from terrestrial vertebrate microbiota (such as humans, rodents, and poultry) and that they do not cover the entire microbial composition of the penguin microbiota. As the 16S rRNA pyrosequencing amplifies all bacterial DNA within the V2–V3 bacterial region, it is likely to provide a more accurate coverage of the bacteria present.

The dominance of Bacteroidetes and Firmicutes at phylum level in penguins is in accordance with previous studies on other vertebrate species, including many mammalian species (Lan et al. 2002; Eckburg et al. 2005; Guo et al. 2008; Ley et al. 2008a,b; Glad et al. 2010a,b; Suenaga 2012). Members of the phylum Firmicutes are associated with the breakdown of complex carbohydrates, polysaccharides, sugars, and fatty acids, which are then utilized by the host as an energy source (Flint et al. 2008; Tap et al. 2009) with high abundances of Firmicutes being linked to adiposity. Although the penguin microbiota in this study is similar to that of other vertebrate species (i.e., dominance of Firmicutes and Bacteroidetes), they do differ from other vertebrates with regard to the relatively high abundance of Fusobacteria (2–55%) and Proteobacteria (5–30%). The composition of the fecal microbiota varies among all four penguin species, with differences observed at both the phylum and family level. These results indicate that there are significant variations within the microbial composition of different species of penguin indicating that the use of a different primer for king penguins did not influence the overall result. While the cause of this variation remains unknown, possible factors, such as diet, prey-associated microbiota, phylogenetic differences, and external environment, have all been found to influence a host microbial composition (Ley et al. 2008a,b; Banks et al. 2009; Roeselers et al. 2011; Nelson 2012). In Adélie penguins, Banks and colleagues (Banks et al. 2009) identified that host phylogeny had a greater influence over host microbial composition in comparison with geographical location. Similarly, Ley et al. (2008a,b) noted that host phylogeny and diet appear to majorly influence the composition at a species and population level, observing distinct differences between herbivores, omnivores, and carnivores. In Antarctic pinnipeds, Nelson (2012) identified that not only did diet and host phylogeny influence the GI microbiota but also that prey-associated microbiota dominated the GI microbiota of the higher predator.

The pyrosequencing data from this study have identified a significant proportion of “unclassified” bacteria, indicating penguins harbor a large unclassified group of resident bacteria in their digestive systems. Earlier studies in terrestrial vertebrates have discovered that previously “unclassified” bacteria to be responsible for important metabolic, immunological, or physiological functions (Pope et al. 2011; Zhu et al. 2011). These bacteria could have the potential to be involved in significant functions relating to digestive physiology (synthesis and storage of proteins, lipids, polyunsaturated fatty acids), health, and disease in penguins and need to be analyzed further.

The 16S rRNA pyrosequencing analysis also highlighted the presence of known pathogens, such as Campylobacter, Helicobacter, Prevotella, Veillonella, Streptococcus, Erysipelotrichaceae, Neisseria, Ureaplasma, and Mycoplasma. The identification of these pathogens is of significant concern due to their potential impact on wildlife health and survival. Although these are known pathogens in humans and other vertebrates, there are limited data available linking these pathogens to disease except for a member of the family Erysipelotrichaceae, which was associated with the death of a little penguin in captivity (Boerner et al. 2004). Also, *Campylobacter* spp. have previously been identified in many penguin species; however, there have been no studies linking the presence of this bacterium to disease in sub-Antarctic penguins (Broman et al. 2000; Bonnedahl et al. 2005; Leotta et al. 2006a,b; Griekspoor et al. 2009). In addition, *Helicobacter* spp. are widely distributed among many vertebrate species including marine mammals (i.e., pinnipeds, cetaceans) (Oxley and McKay 2005; Goldman et al. 2009), and although some species have been associated with disease in humans and some marine mammals (Harper et al. 2000; Oxley and McKay 2005), there are no data linking the presence of Helicobacter to illness or disease in marine seabirds. The presence of these pathogens may also indicate transmission from humans, which needs further investigation.

Because of their dense colonial living, penguins are more susceptible to rapid pathogen transfer, which could lead to major disease outbreaks (Griekspoor et al. 2009). Therefore, the presence of any known pathogen warrants further investigation to ascertain if these organisms are in fact pathogenic to penguins, and if so, what would the potential impact be on the population.

In summary, this study has identified that there is large variation within the fecal microbiota of sub-Antarctic and temperate penguin species and that these microbiota appear to be dominated by five major phyla: Firmicutes, Bacteroidetes, Proteobacteria, Fusobacteria, and Actinobacteria. Although the cause of these differences is yet to be determined, host phylogeny and diet could potentially play a major role in determining the final microbial composition of an individual. This study also identified the presence of known mammalian pathogens that could potentially cause illness or disease within a penguin population and these findings warrant further investigation.

## Supporting Information

**Figure S1.** Actinomycetaceae is the mist dominant family within the phylum Actinobacteria in king, little, and macaroni penguins but its abundance was relatively low in gentoo penguins. While Corynebacteriaceae and Propionibacteriaceae are the most dominant families in gentoo penguins.

**Figure S2.** Porphyromonadaceae is the most abundant family within the phyla Bacteroidetes in all penguin species. Flavobacteriaceaaea also dominates the phylum Bacteroidetes in gentoo penguins.

**Figure S3.** The fmaily composition within the phylum Firmicutes is significantly different in all penguin species. For gentoo and macaroni penguins, Ruminococcaceae is the dominant family within the phylum Firmicutes. In king penguins Clostridiales family XI is the dominant family, while in little penguins Lactobacilliaceae.

**Figure S4.** There are significant differences within the family composition of the phylum Proteobacteria in all penguin species. In gentoo and macaroni penguins, Moraxellaceae dominates the phylum Proteobacteria. In king penguins Campylobacteriaceae is the most dominant family, while in little penguins, Enterobacteriaceae is the most dominant.

**Table S1.** Quantitative real-time PCR primer sequences used in this study to detect and quantify major phyla present in penguin fecal samples.

**Table S2.** Chi-squared analysis of statistical difference between little, gentoo, and macaroni penguins.

**Table S3.** Taxonomic assignment of sequence reads of all penguin species.
