# Supplementary material for: Interspecific variations in the gastrointestinal microbiota in penguins
Source: Microbiologyopen. 2013 Jan 25;2(1):195–204. doi: 10.1002/mbo3.66 (PMC3584224; doi:10.1002/mbo3.66)
Supplement: Supplementary file 1 [file mbo30002-0195-SD1.doc]

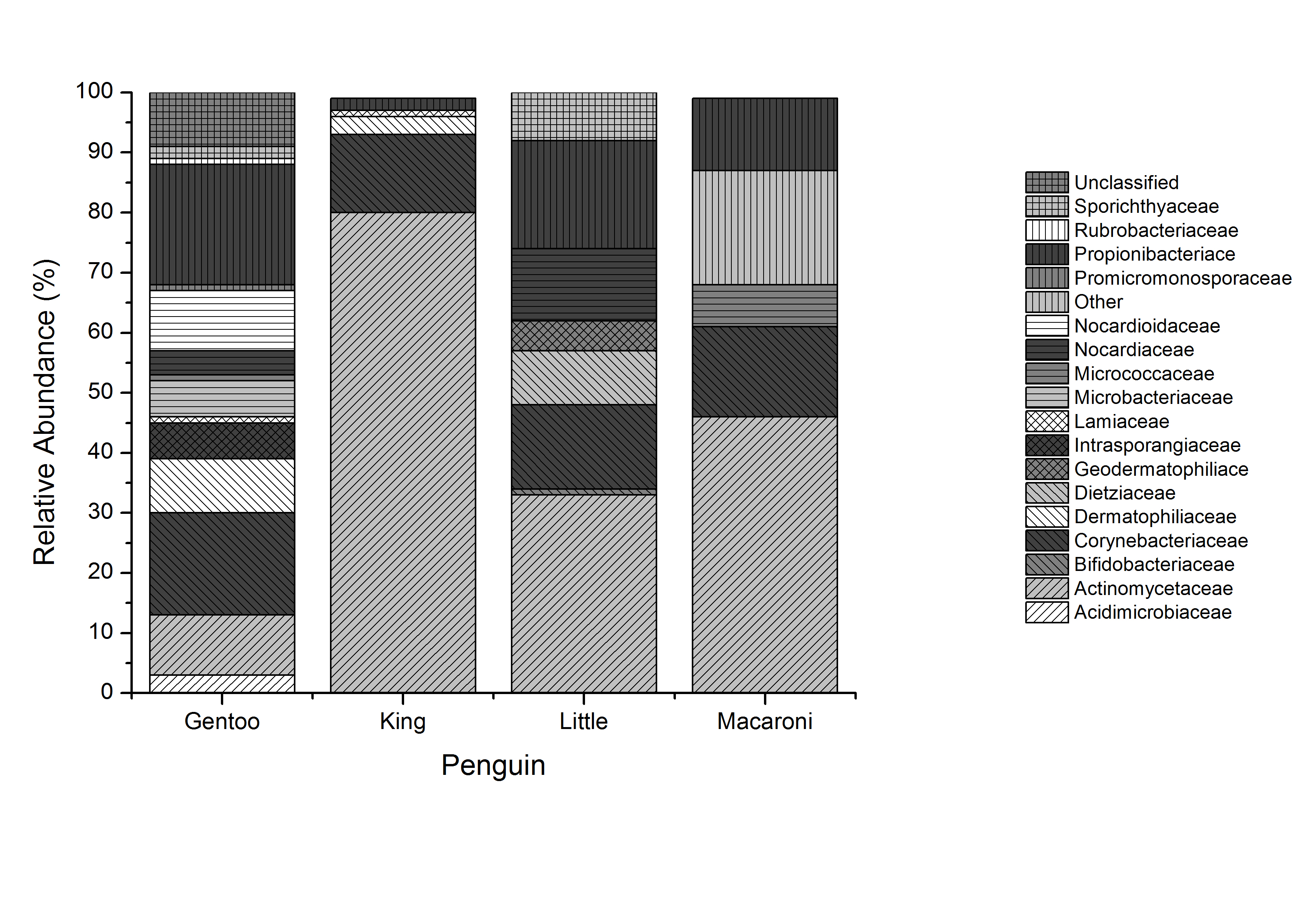


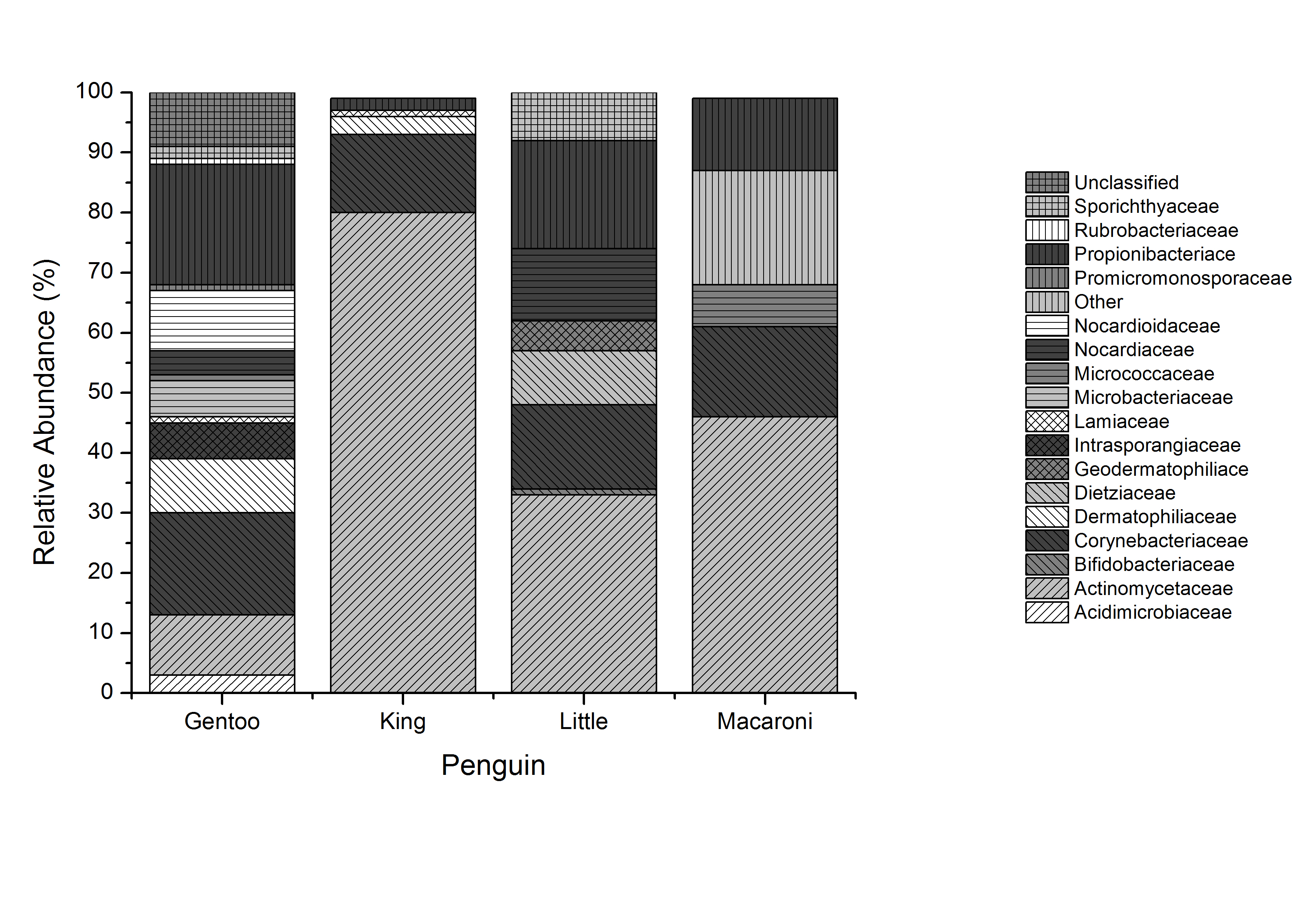


**Figure S1,** Actinomycetaceae is the mist dominant family within the phylum Actinobacteria in king, little and macaroni penguins but its abundance was relatively low in gentoo penguins. While Corynebacteriaceae and Propionibacteriaceae are the most dominant families in gentoo penguins.
