# Supplementary material for: Interspecific variations in the gastrointestinal microbiota in penguins
Source: Microbiologyopen. 2013 Jan 25;2(1):195–204. doi: 10.1002/mbo3.66 (PMC3584224; doi:10.1002/mbo3.66)
Supplement: Supplementary file 2 [file mbo30002-0195-SD2.doc]

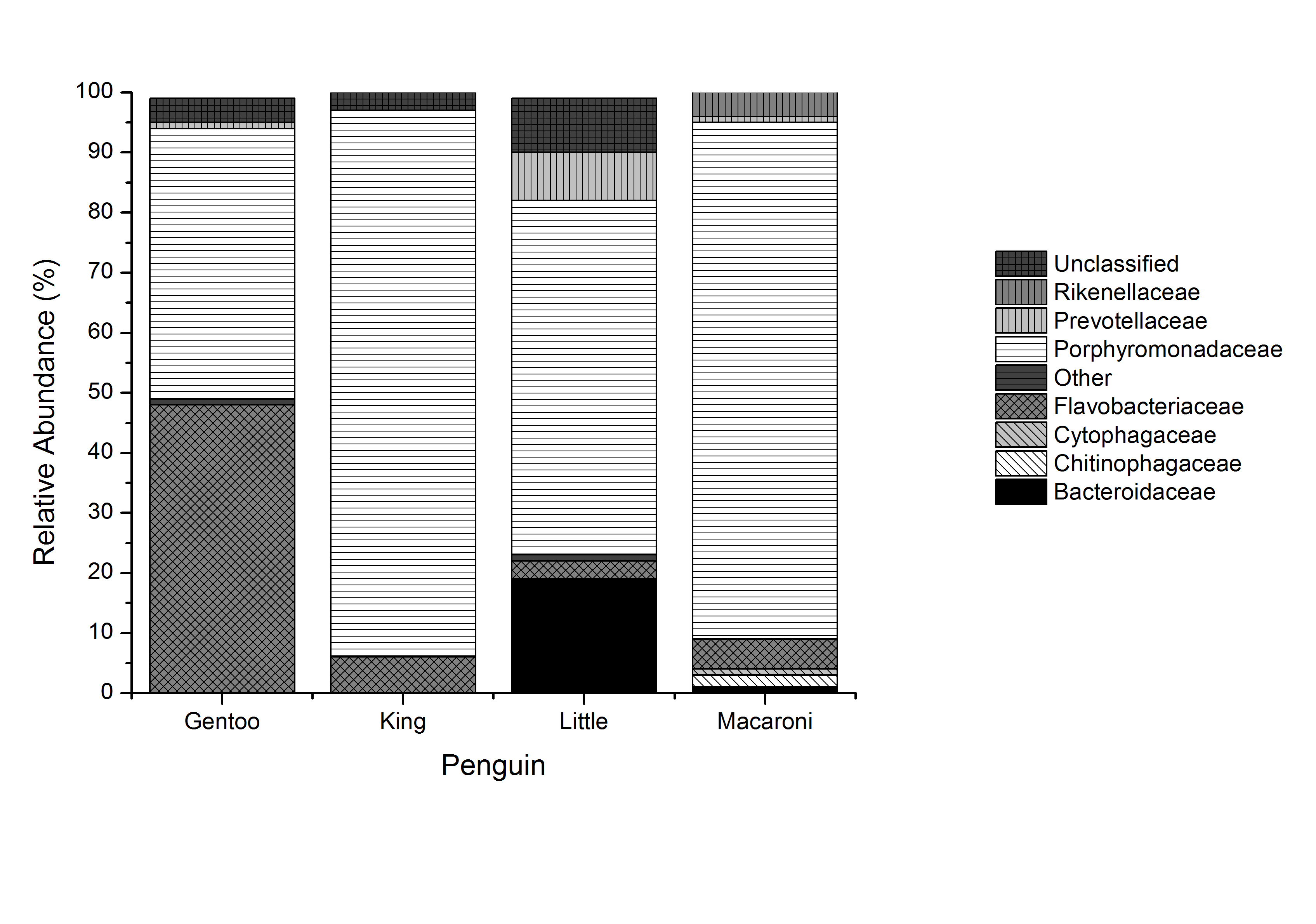


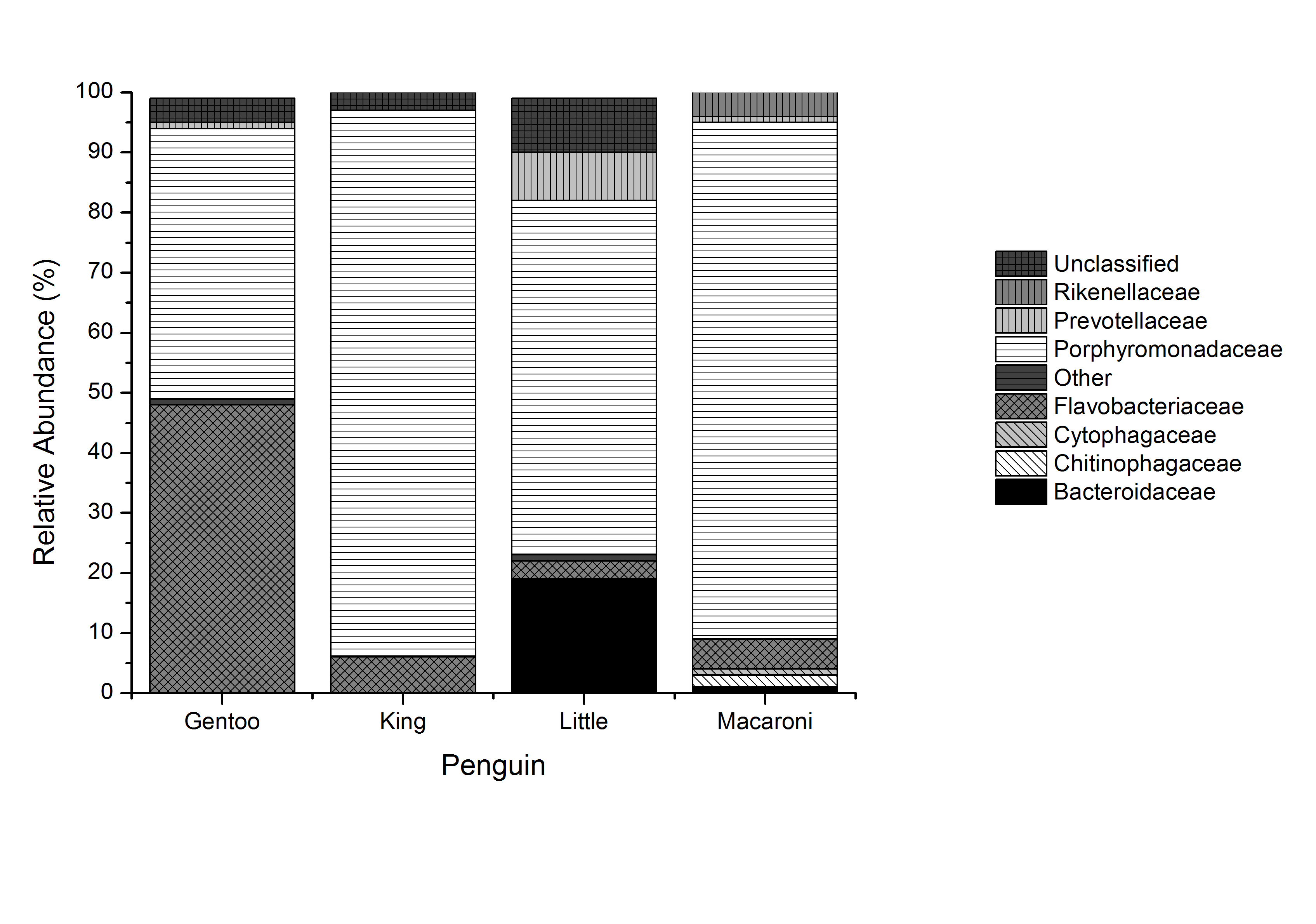


**Figure S2,** Porphyromonadaceae is the most abundant family within the phyla Bacteroidetes in all penguin species. Flavobacteriaceaaea also dominates the phylum Bacteroidetes in gentoo penguins.
