# Supplementary material for: Interspecific variations in the gastrointestinal microbiota in penguins
Source: Microbiologyopen. 2013 Jan 25;2(1):195–204. doi: 10.1002/mbo3.66 (PMC3584224; doi:10.1002/mbo3.66)
Supplement: Supplementary file 3 [file mbo30002-0195-SD3.doc]

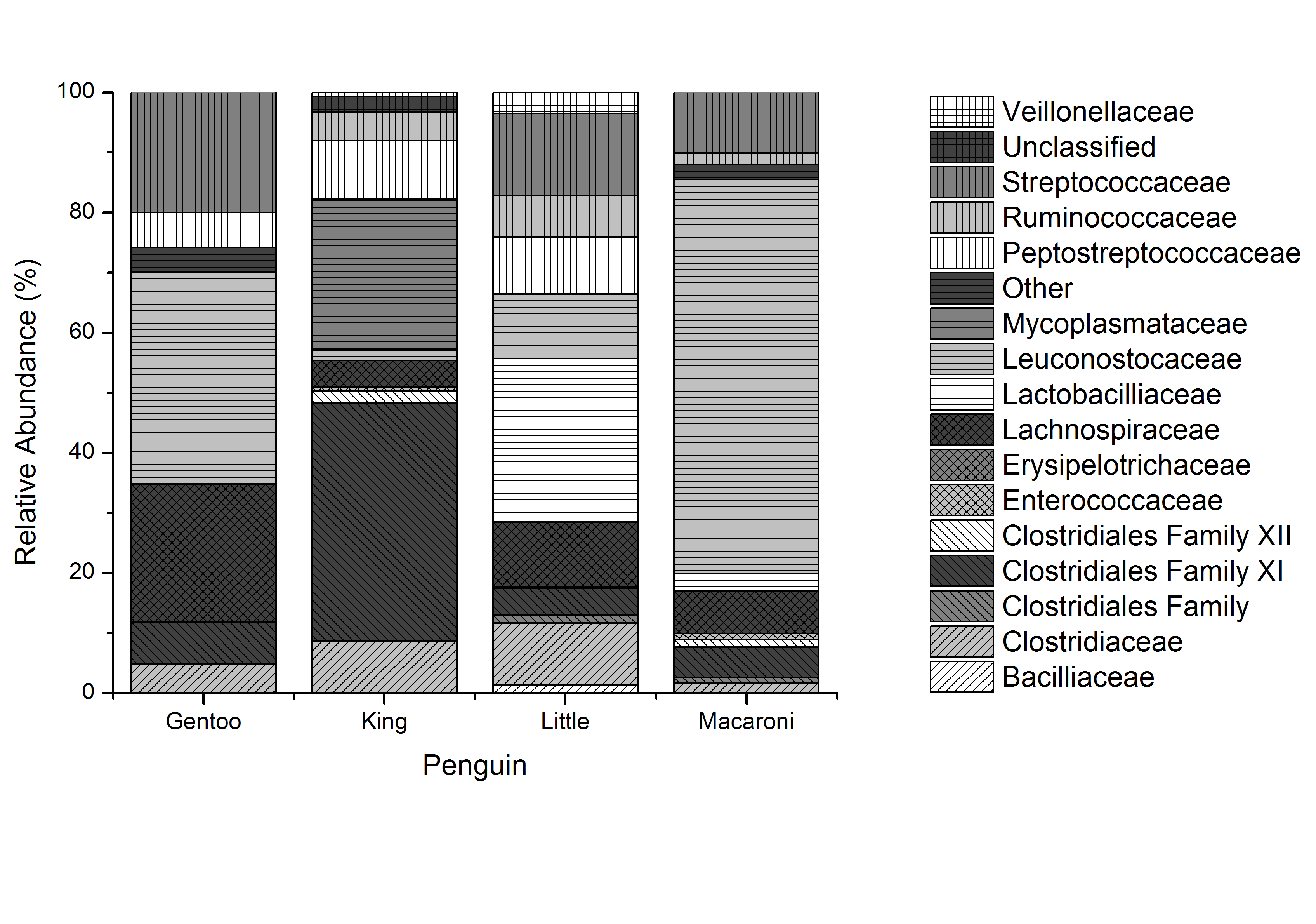


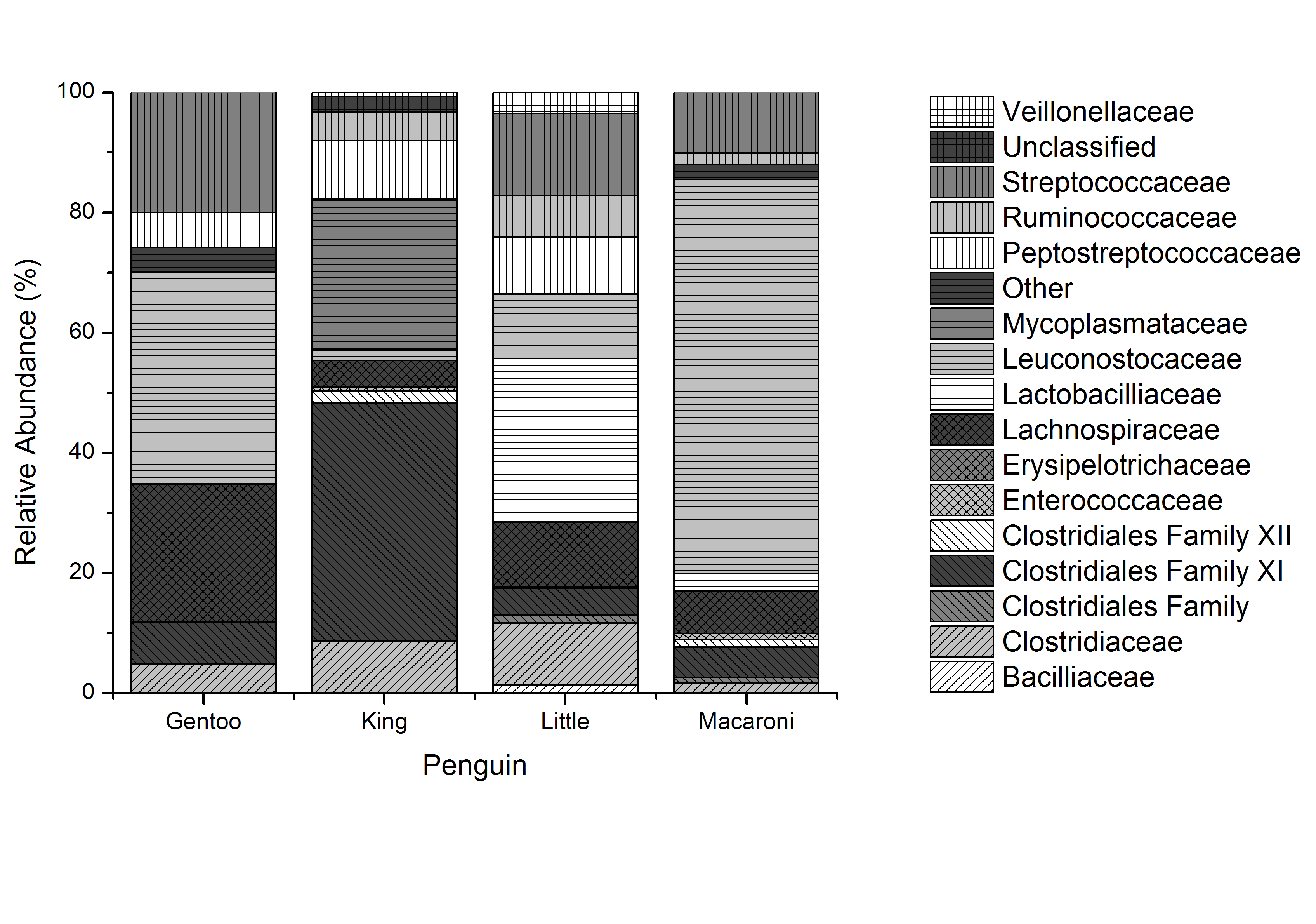


**Figure S3,** The fmaily composition within the phylum Firmicutes is significantly different in all penguin species. For gentoo and macaroni penguins, Ruminococcaceae is the dominant family within the phylum Firmicutes. In king penguins Clostridiales Family XI is the dominant family, whilst in little penguins Lactobacilliaceae.
