# Supplementary material for: Interspecific variations in the gastrointestinal microbiota in penguins
Source: Microbiologyopen. 2013 Jan 25;2(1):195–204. doi: 10.1002/mbo3.66 (PMC3584224; doi:10.1002/mbo3.66)
Supplement: Supplementary file 4 [file mbo30002-0195-SD4.doc]

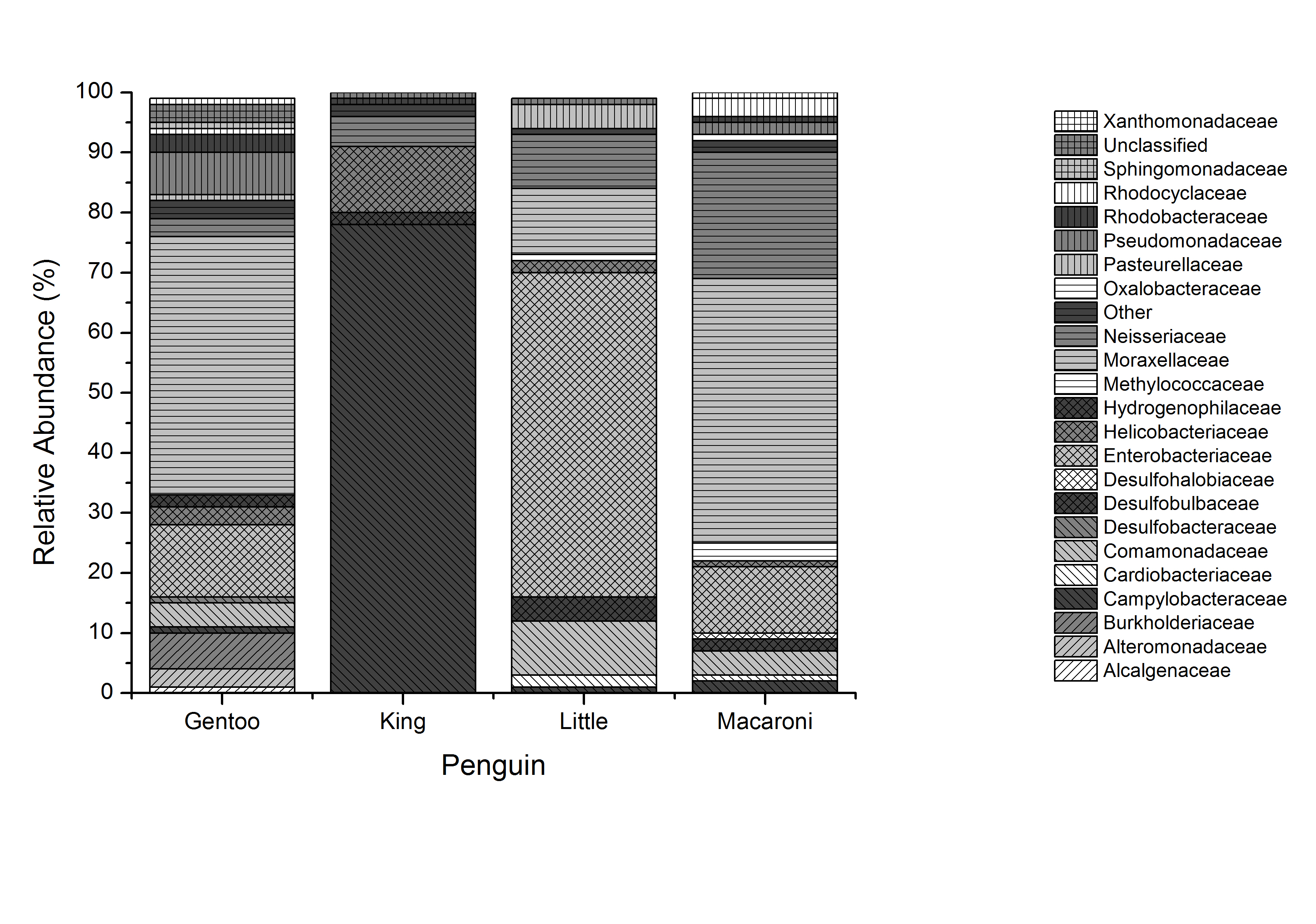


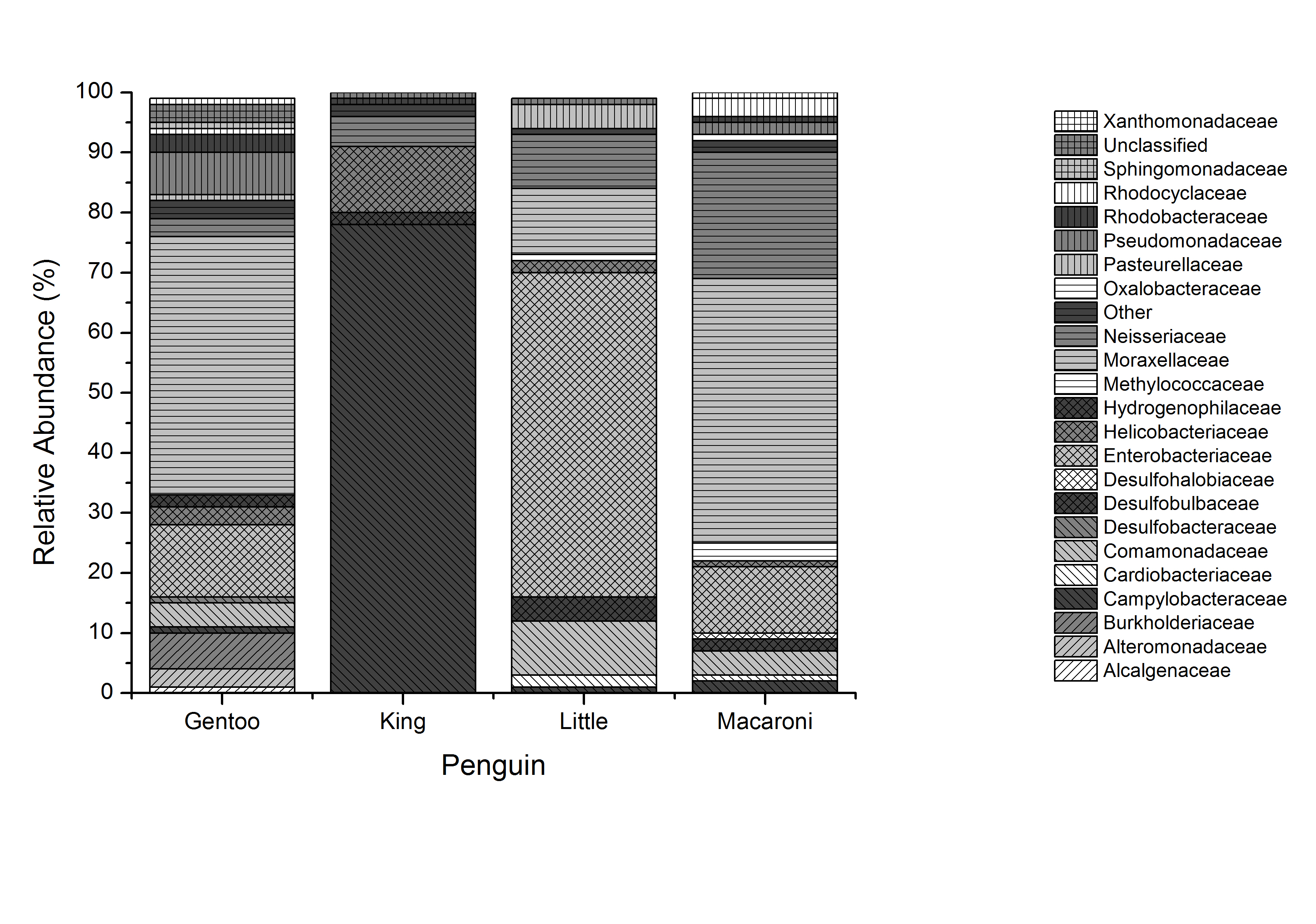


**Figure S4,** There are significant differences within the family composition of the phylum Proteobacteria in all penguin species. In gentoo and macaroni penguins, Moraxellaceae dominates the phylum Proteobacteria. In king penguins Campylobacteriaceae is the most dominant family, whilst in little penguins, Enterobacteriaceae is the most dominant.
