# Supplementary material for: Interspecific variations in the gastrointestinal microbiota in penguins
Source: Microbiologyopen. 2013 Jan 25;2(1):195–204. doi: 10.1002/mbo3.66 (PMC3584224; doi:10.1002/mbo3.66)
Supplement: Supplementary file 5 [file mbo30002-0195-SD5.doc]

| **Phylogenetic Target** | **Primer** | **Sequence** | **Reference** |
| --- | --- | --- | --- |
| Firmicutes | Firm 350  Firm 815 | GGCAGCAGTRGGGAATCTTC  ACACYTAGYACTCATCGTTT | Mϋhling et al 2008 |
| Bacteroidetes | 934  1060 | GGARCATGTGGTTTAATTCGATGAT  AGCTGAACGACAACCATGCAG | Guo et al 2008 |
| Actinobacteria | Eub338F  Act1159R | ACGGGCGGTGTGTACA  TCCGAGTTRACCCCGGC | Blackwood et al 2005 |
| Alphaproteobacteria, Deltaproteobacteria Fusobacterium | ADF681F  1392R | AGTGTAGAGGTGAAATT  ACGGGCGGTGTGTACA | Blackwood et al 2005 |
| Betaproteobacteria | Beta680F  1392R | CTCGTGTAGCAGTGA  ACGGGCGGTGTGTACA | Blackwood et al 2005 |
| Gammaproteobacteria | Gamma395f  Gamma871r | CMATGCCGCGTGTGTGAA  ACTCCCCAGGCGGTCDACTTA | Mϋhling et al 2008 |
| Epsilonproteobacteria | Epsilon F  Epsilon R | AGC GTT AYT CGG AAT CAC TGG  CCC CGT CTA TTC CTT TGA GTT TT | Richberg 2000 |

Table S1, Quantitative real time PCR primer sequences used in this study to detect and quantify major phyla present in penguin faecal samples


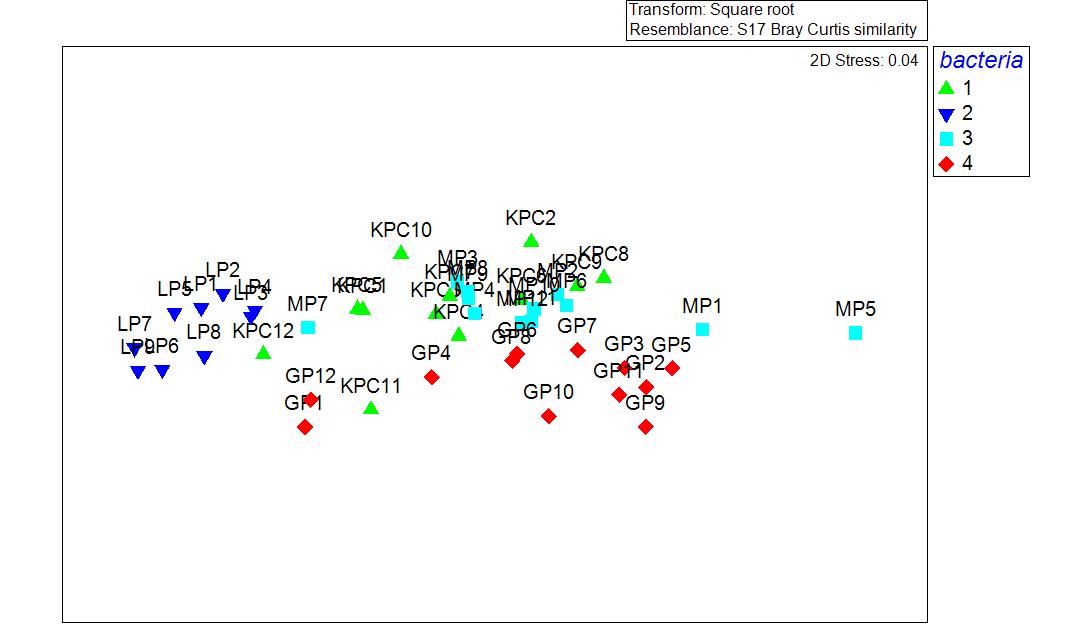


**Figure S1**. MDS graph of quantitative real time PCR data for king, gentoo, little and macaroni penguins
